# Mapping Mental Representations With Free Associations: A Tutorial Using the R Package associatoR

**DOI:** 10.5334/joc.407

**Published:** 2025-01-06

**Authors:** Samuel Aeschbach, Rui Mata, Dirk U. Wulff

**Affiliations:** 1 Center for Adaptive Rationality, Max Planck Institute for Human Development, Berlin, Germany; 2 Center for Cognitive and Decision Sciences, University of Basel, Basel, Switzerland

**Keywords:** free association, mental representations, intelligence, generative AI, R package

## Abstract

People’s understanding of topics and concepts such as risk, sustainability, and intelligence can be important for psychological researchers and policymakers alike. One underexplored way of accessing this information is to use free associations to map people’s mental representations. In this tutorial, we describe how free association responses can be collected, processed, mapped, and compared across groups using the R package *associatoR*. We discuss study design choices and different approaches to uncovering the structure of mental representations using natural language processing, including the use of embeddings from large language models. We posit that free association analysis presents a powerful approach to revealing how people and machines represent key social and technological issues.

Free association involves asking people to produce—in oral or written form—the words that come to mind when they are presented with a cue word or phrase. It offers several advantages over other psychological research methods, including ease of use, facilitation of spontaneous responses, redundancy of experimenter-defined response options, and potential to explore heterogeneity in human responses. Analysis techniques for free association research have advanced significantly of late due to progress in natural language processing, and are not yet well known to researchers in psychology. This tutorial aims to overcome this barrier by guiding researchers through the analysis of free association data using the *associatoR* package for the open-source programming language R (Aeschbach & Wulff, 2023). Our aim is to increase the accessibility of free association analysis and help researchers leverage its potential for diverse research questions in the cognitive and social sciences.

## Mapping Mental Representations With Free Associations

Free association has a rich history in psychology, going back to the 19th century when Francis Galton used it as a means to understand how individual experiences shape thinking and memory (Galton, 1883). Since then, free association methods have been adopted in several research areas, including clinical psychology (Jung, 1910), implicit attitudes (Schnabel & Asendorpf, 2013), and memory (Nelson et al., 2000).

Much work using free associations has focused on understanding the structural characteristics of mental representations. For example, the large-scale citizen science project Small World of Words (SWOW; De Deyne et al., 2019) and similar projects (Kiss et al., 1973; Nelson et al., 2004; Steyvers & Tenenbaum, 2005) have studied key properties of semantic networks, such as their small-world organization, with high clustering and short path lengths (Steyvers & Tenenbaum, 2005). Data from such studies have been instrumental in understanding individual and age differences in the structural characteristics of semantic networks (Wulff et al., 2019). For example, this research has revealed systematic differences between the semantic networks of younger and older adults, with older adults’ networks reliably being less dense, clustered, and efficient (Dubossarsky et al., 2017; Wulff, Aeschbach, et al., 2022; Wulff, De Deyne, et al., 2022; Wulff, Hills, & Mata, 2022). Other efforts have focused on investigating individual and group differences in the structure of semantic networks with respect to intelligence and creativity (e.g., Kenett et al., 2014). Overall, this work highlights how free associations, paired with modern analytic approaches such as network analysis, can significantly advance the understanding of human cognition.

Other research has used free association to reveal people’s knowledge and attitudes about central societal topics or concepts (Koll et al., 2010; Szalay & Brent, 1967; Zaller & Feldman, 1992). For example, recent work has explored lay concepts of sustainability (Barone et al., 2020), artificial intelligence (Selwyn & Gallo Cordoba, 2022), and risk (Wulff & Mata, 2022). Using free associations to map people’s semantic representation of the concept of risk, Wulff and Mata (2022) uncovered dimensions not previously considered in the academic literature and highlighted the importance of age and gender differences in the understanding of various societal risks. This work demonstrates how free associations can reveal individual variation in people’s thinking about various topics in ways that overcome the limitations of other approaches, such as surveys or interviews. Whereas traditional closed-form survey items must be predefined by researchers and can exhibit strong demand characteristics, free associations are bottom-up, allowing participants to share what they think about a topic independently of the researcher’s perspective (Avis et al., 2014; Wulff & Mata, 2022). This critically includes a natural assessment of importance based on what participants do or do not retrieve. Moreover, free associations are much less labor-intensive than interviews, allowing researchers to work with larger samples and produce data that are readily amenable to quantitative analysis. While future work must further clarify the method’s benefits and limitations, there is clearly untapped potential for the cognitive and social sciences to investigate people’s knowledge and attitudes using free associations.

## A Guide to Analyzing Free Associations

This article presents a practical guide to using free associations to map aggregate patterns and explore group and individual variation in mental representations, focusing on specific topics or concepts. First, we introduce the reader to free association studies and the data they produce. Second, we provide a tutorial on how to analyze these data, from preprocessing to the final stages of generating insights by visualizing associations and making comparisons between groups. To this end, we utilize the *associatoR* package (Aeschbach & Wulff, 2023) for the open-source programming language R (R Core Team, 2023), which was specifically built to simplify the processing and analysis of free association data.

### Free Association Studies and Data

**Figure 1:**
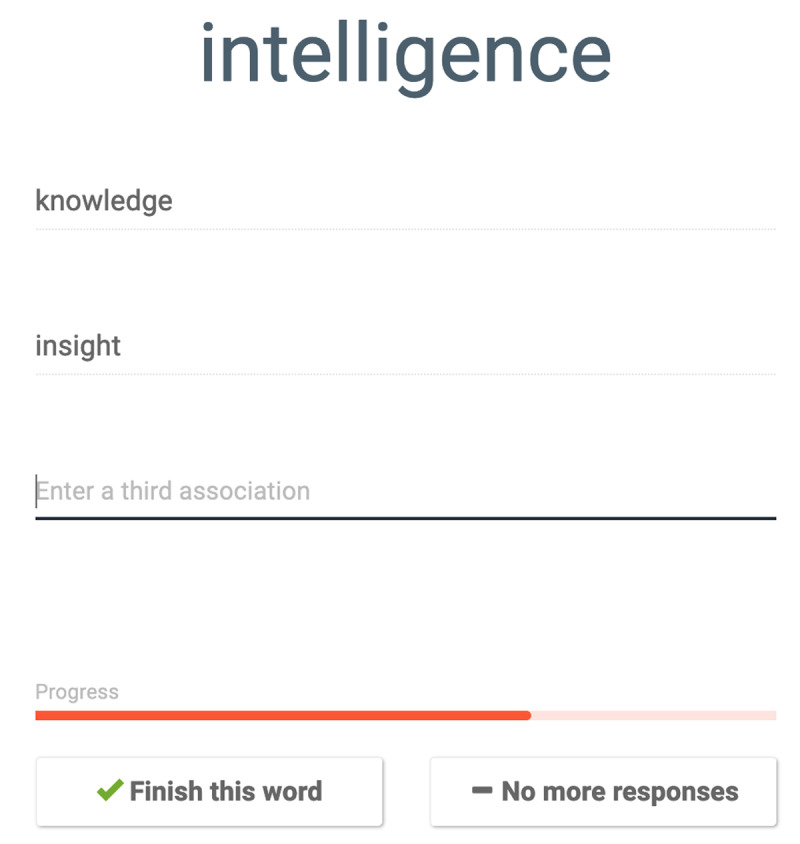
Free association user interface of the Small World of Words study (https://smallworldofwords.org) showing the cue word ‘intelligence’ at the top. Below are three lines for respondents to enter free associations, with two already populated by the response words ‘knowledge’ and ‘insight.’

Free association studies ask participants to generate one or more associates to one or more cues. For example, the Small World of Words (SWOW) study has asked thousands of online volunteers to generate up to three associates to a small subset of the thousands of cues included in SWOW (see Figure 1; De Deyne et al., 2019). Free association studies can, however, vary in several ways. For example, whereas studies like SWOW seek to map associates to a large set of cues, other free association studies may be interested in a specific cue (e.g., artificial intelligence; Selwyn & Gallo Cordoba, 2022). Furthermore, whereas a single response to each cue was traditionally collected (e.g., Nelson et al., 2004), recent studies have tended to use continued association tasks, collecting multiple responses to each cue, to elicit a greater diversity of associations (e.g., De Deyne et al., 2013). In addition, studies may differ in the constraints posed on the production of associates, such as imposing a time limit. Other variations concern the mode of response (typed or verbal), as well as other design aspects, such as the use of snowball techniques, in which a previously generated response becomes the cue for additional follow-up responses.

Regardless of the study design, the data generated will typically include a set of participant-, cue-, and response-related information. The relative amount of information for each category will vary from study to study as a function of the specific study design and research questions addressed.

**Figure 2:**
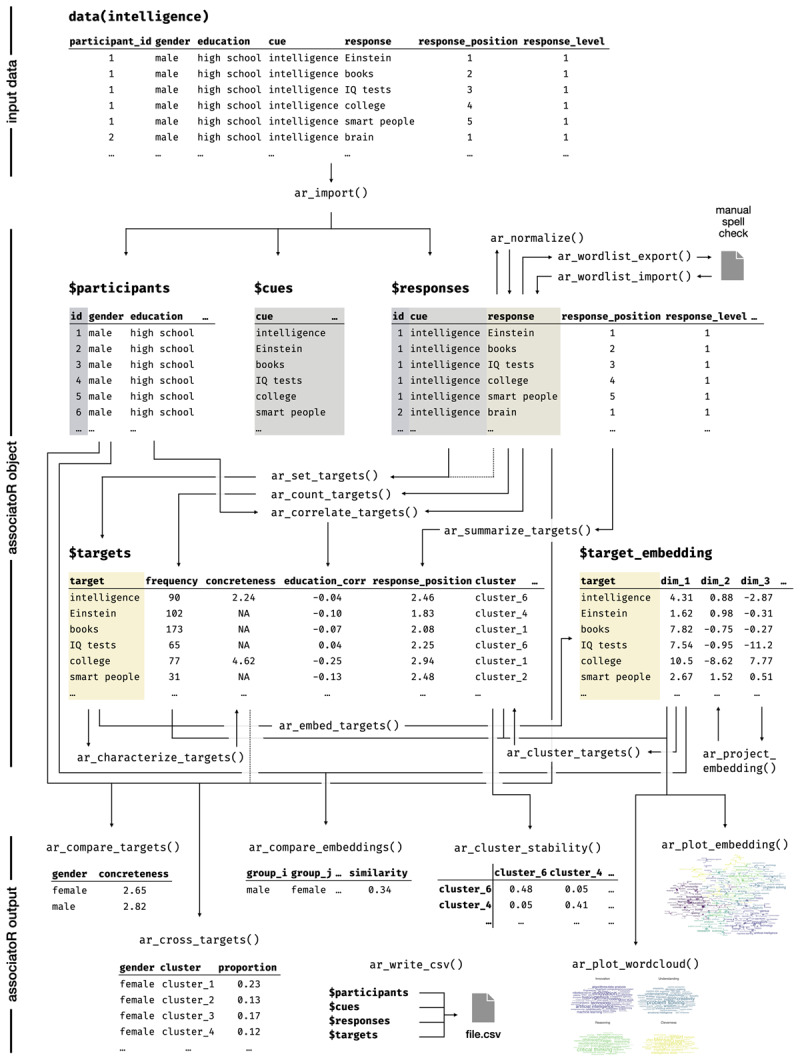
Overview of the working example with associatoR functions and class ‘associatoR’ object.

To help readers familiarize themselves with the *associatoR* package, we have created synthetic data representing the outcome of a typical free association study. This working example data, displayed at the top of Figure 2, was generated to match the design of a recent study analyzing the public’s semantic representation of a specific psychological construct (‘risk’; Wulff & Mata, 2022). This study collected five first-level free association responses to a top-level cue (‘risk’), followed by five second-level associations to each of the first-level responses, giving a total of 30 associations per participant. The rationale of this mini-snowball paradigm was that associations to the second-level responses could be used to characterize the relationships between associations to the top-level cue (‘risk’). The original sample was stratified by age and gender, making it possible to compare the representation of the construct across different demographic groups.

In our working example, we adopted the same mini-snowball design as Wulff and Mata (2022), and focused on another central construct in cognitive psychology: intelligence. Investigating the mental models underlying this construct (Coane et al., 2023; Sternberg et al., 1981) can help to bridge gaps between expert and public understandings of the construct, and facilitate comparisons between human and machine intelligence (Cave & Dihal, 2019). For our demonstration purposes, we generated 1,000 synthetic participants using a large language model, GPT-4-Turbo (gpt-4-0125-preview; OpenAI, 2023), and used prompting to introduce group variation, creating four demographic groupings that varied in gender (*male* versus *female*) and level of education (*high school* versus *university*). This data set is made available with the R package as an object named intelligence. More details behind the generation of the data, including the prompts used, are presented in the Appendix and the package documentation (accessible through ?intelligence).

### An overview of *associatoR*

The *associatoR* package was built to streamline the analysis of free association data, focusing on applications that reveal mental representations of specific topics and concepts. Figure 2 provides an overview of central functions, the associatoR class object, and workflows based on our working example. The package can be installed from GitHub and loaded into R using the code below. All following code assumes that the *associatoR* package has been installed and loaded into the current R session.

**Figure:**
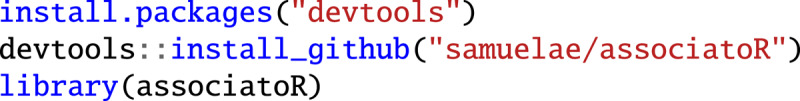

Using the *associatoR* package can be broken down into seven steps. First, data are imported using ar_import(), which creates the class associatoR object consisting of the participants, cues, and responses tables. Second, the data are preprocessed to improve subsequent analysis by normalizing and correcting spellings. Third, some of the imported data, cues, and/or responses are set as the analysis target, creating the targets table, which plays a pivotal role within the associatoR architecture. Fourth, targets are embedded, creating the target_embedding table, which facilitates the clustering and mapping of targets. Fifth, the targets are characterized based on embedding clusters, psycholinguistic norms, and other data. Sixth, visualizations are generated using the target embeddings and characteristics that illustrate response distributions. Seventh, target characteristics are compared based on participant characteristics. In the following, we introduce these steps and demonstrate how to implement them for our working example using the *associatoR* package.

## 1. Importing data

The first step in using the *associatoR* package is to import the free association data into the associatoR object, which is the required input for all *associatoR* functions. The associatoR object breaks the input data down into three tables that provide a flexible basis for subsequent analyses: a participants table, a cues table, and a responses table. The participants table contains variables that characterize participants (here, gender and education); the cues table contains variables that characterize cues, such as cue category or sentiment (in this case, none); the responses table contains the actual free association response data, including variables characterizing the responses, such as response position or sentiment (here, response position and level).

### Importing data with *associatoR*

The ar_import() function imports data into associatoR. It takes an R data frame as input and requires that the names of at least three variables are identified: the variable containing the participant id (participant), the cue words or phrases (cue), and the association responses (response). Optionally, additional variables with information on the participants (participant_vars), cues (cue_vars), and responses (response_vars) can be specified. Note how *associatoR* uses lazy evaluation, permitting the use of variable names without quotation marks. The ar_import() function returns an R list of class associatoR that is the expected input of all subsequent *associatoR* functions.

**Figure:**
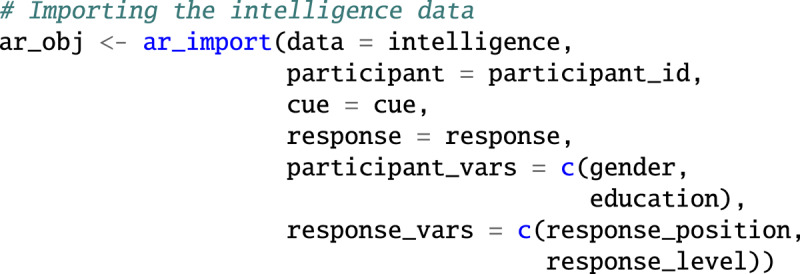

## 2. Preprocessing data

Like other forms of qualitative assessment, free association responses require preprocessing to facilitate subsequent analyses. Two steps that typically improve free association analysis are normalizing and spelling correction. Normalizing removes aspects of word forms usually regarded as irrelevant to meaning, such as capitalization, white spaces, or punctuation. Consider the word forms ‘banana’, ‘Banana’, ‘BANANA’, and ‘ banana’. Participants thought about the same fruit in each case, but wrote the word differently, probably for arbitrary reasons. Normalizing these word forms by unifying the casing of the letters and removing superfluous white spaces helps to avoid an unnecessary proliferation of word forms and to produce response frequencies that better reflect respondents’ intention. In our working example generated with GPT-4, normalization had a small effect, but it can significantly alter human-generated data. It is thus always important to consider the goals of the investigation and whether normalization can further these goals.

Spelling correction implies using a dictionary to reconstructing the correct spelling of a word. Like variation in capitalization and white spaces, misspellings can result in multiple word forms for one and the same intended response. While correcting these word forms is recommended, doing so can be tricky, as spelling correction is an inferential process that can introduce errors.

Two further preprocessing steps are lemmatization and stemming. These steps result in further homogenization of responses by assigning the same word forms to words with the same base lemma or word stem. This reduces the total number of word forms, which can simplify but also distort analyses. For instance, plural responses are changed to their singular form or word stem, which can imply a loss of nuance when the distinction between singular and plural is relevant to the analysis. Generally, it is recommended to keep processing to a minimum, focusing on normalization and spelling correction, and avoiding more invasive forms of processing, such as lemmatization and stemming.

### Data preprocessing with *associatoR*

The ar_normalize() function normalizes word forms in three ways. First, based on the setting of the case argument, it changes the letter case to lower case (“lower”), upper case (“upper”), sentence case (“sentence”), or to the most frequent letter case among the responses (used below, “most_frequent”). Second, based on the setting of the punct argument, it removes either all punctuation (“all”, used below) or only punctuation at the end of the string (“end”). Based on the setting of the whitespace argument, it removes all white spaces at the beginning and end of the string (“trim”) or additionally duplicated white spaces within the string (“squish”). Finally, because the process_cues argument is set to TRUE, it applies the normalization to responses and cues. The code below illustrates how to apply the ar_normalize() function to ar_obj, containing the working example. To perform additional normalization, *associatoR* also includes the ar_normalize_manual() function, which can be supplied with processing functions from other text-processing packages, such as *stringr* (Wickham, 2023), *udpipe* (Wijffels, 2023), or *tm* (Feinerer et al., 2008).

**Figure:**
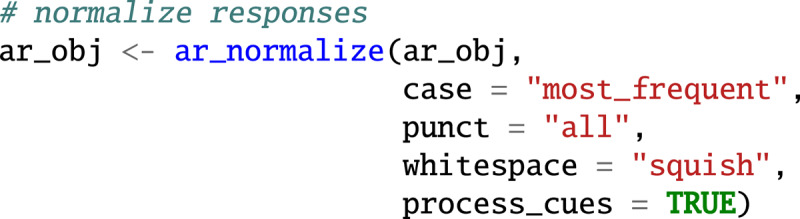

The *associatoR* package does not provide functions for automatic spellchecking due to it being an error-prone process, especially when words and phrases lack a strong textual context, as is the case for free associations. However, *associatoR* does facilitate manual spellchecking, which we recommend. Specifically, it offers two functions, ar_wordlist_export() and ar_wordlist_import(). The ar_wordlist_export() function generates a table of unique association responses with three columns: response, containing the original response form, response_correct, providing a placeholder for corrections based on the manual spellcheck, and response_frequency, indicating the frequency of the response form among all responses as a rough indicator of correctness—misspelled responses are typically low in frequency. The ar_wordlist_export() function takes the associatoR object as input; it additionally requires a file path (file) for the location of the wordlist table, and optionally a string specifying whether to sort alphabetically or based on response frequency (sort). Following manual completion of the file exported by ar_wordlist_export(), the file can be imported back into associatoR to apply the corrections to the associatoR object using the ar_wordlist_import() function. It takes the associatoR object and the file path as inputs, as well as an additional argument apply_to, which can be “responses” or “all” to correct the responses or both responses and cues.

**Figure:**
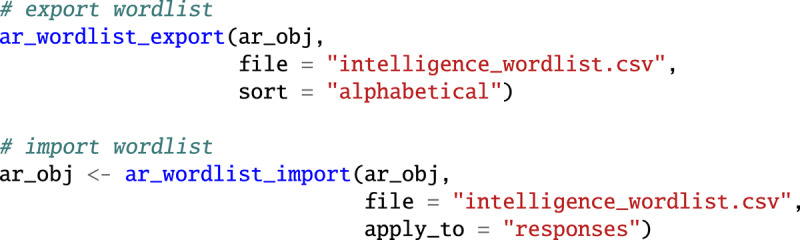

## 3. Setting targets

A pivotal step in the analysis of free associations is to decide on the targets of the analysis, which can be either cues or cues and responses. Which to choose depends on the study design and research question. In the working example, modeled after the study by Wulff and Mata (2022), associations were generated at two levels: (1) Associations were first generated for the top-level cue, ‘intelligence.’ (2) The first-level responses were then used as cues to generate second-level responses. In our analysis, the cues, which include both the top-level cue and the first-level responses, are of primary interest. The second-level responses can be thematically quite distant from the top-level cue, and thus contribute less to revealing the semantic representation of the top-level cue. If the goal is to understand the semantic representation of a particular topic, as is the case in our working example, and second- or higher-level responses are present, targets would be set to cues only. This will be different in other studies, where second- or higher-order responses may not be available, or where the goal of the study is to map semantic representations more generally (e.g., De Deyne et al., 2013; Morais et al., 2013). In these settings, it makes sense to set the targets to both cues and responses.

This important conceptual aspect of free association studies is reflected in the architecture of *associatoR* functions. In *associatoR*, the targets are set once at the beginning of the analysis, to the effect that all subsequent analyses are constrained to the specified set of targets. In some cases, this determines the types of analyses available to the user. For instance, in Section 4, we use the second-level responses to construct target embeddings, which would not be possible if responses were included in the targets.

### Setting targets with *associatoR*

The function ar_set_targets() sets the analysis targets. It takes the associatoR object as input and requires specification of the targets argument, which determines whether targets are populated based on cues (“cues”), responses (“responses”), or both (c(“cues”, “responses”)). The code below sets the targets to cues only, which is appropriate given the two-level mini-snowball approach used in our working example.

**Figure:**
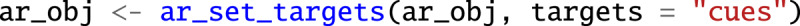

## 4. Embedding targets

Another key preparatory step in free association analysis is embedding the targets. In natural language processing, embedding is a technical term describing a numerical representation that captures the relationships between units of text, in this case, the targets. Embedding facilitates a host of downstream analyses, such as visualizing targets, clustering targets, and comparing mental representations between groups of individuals (see, e.g., Hussain, Mata, & Wulff, 2024; Hussain, Binz, et al., 2024).

There are two major ways to generate target embeddings from free association data. The first is to use the existing data. This is possible when the cues are set as analysis targets, leaving the responses available as a basis to produce a target embedding. Our working example allows for this type of embedding. Extracting an embedding from responses involves tabulating the distribution of (second-level) responses for every target. This produces a matrix consisting of *N* targets and *M* responses, which can be understood as an *M*-dimensional embedding space containing *N* target vectors defined by the *M* response frequencies, whose proximity in space reflects the similarity of response frequencies. Two additional steps further improve the embedding. First, the embedding is normalized using positive point-wise mutual information (PPMI; Bullinaria & Levy, 2007; Church & Hanks, 1990) to reduce the weight assigned to frequent targets and responses. Second, singular value decomposition (SVD) is used to reduce the dimensionality of the embedding, which makes the embedding more manageable and helps infer latent relationships between targets (Bullinaria & Levy, 2012; Richie & Bhatia, 2021). Past work has shown that combining PPMI and SVD produces embeddings that accurately capture human psychology (Hussain, Mata, & Wulff, 2024; Vankrunkelsven et al., 2018).

The second approach to generating a target embedding is to use external data. This can be either other free association data, such as SWOW, which can be used analogously to the second-level responses, or large language models, which can be used to extract target embeddings directly (Hussain, Binz, et al., 2024). Large language models are neural networks trained on large amounts of digitized text, enabling them to accurately interpret and produce text (Bubeck et al., 2023; Hussain et al., 2024; Wulff & Mata, 2023). One powerful use of these models is feature extraction, which involves recording the hidden activations within the model for different inputs and using these activations as embeddings of those inputs. Embeddings produced by feature extraction from large language models have been found to align well with human psychology in many respects (Cassani et al., 2023; Hussain, Binz, et al., 2024). There are now various ways of accessing large language models. One attractive solution, considering principles of reproducible science, is the open-source Hugging Face ecosystem (https://huggingface.co/; see, Hussain et al., 2024). Hugging Face offers access to hundreds of thousands of models that can be run locally or remotely via an API.

The different approaches to producing target embeddings each have their advantages and limitations. The advantage of using an extra response level in the study design is that relationships between targets are contextualized based on the study’s topic and participants. The resulting embeddings thus are likely high in validity. The primary limitation, on the other hand, is low reliability. This especially concerns cues that occur only a few times as first-level responses. In our working example, for instance, 26% of targets occurred three or fewer times. This implies that at most 15 responses are available to identify the embedding location of these targets, which likely will be insufficient to place them reliably in relation to the other targets. This limitation can, in some cases, be addressed by using other free association data, such as SWOW. The public SWOW data of the English project contains 300 responses for each of over 12,000 cues, which is typically sufficient to produce reliable embeddings. Another benefit of using SWOW data is that, in principle, participants could be matched based on demographic characteristics to increase the validity of the data. However, a limitation of using external free association data such as SWOW is that not all targets are covered. Only 37% of the targets in our working example are included in the current SWOW data. This means that extracting complete target embeddings from SWOW is not feasible for our working example or indeed for many other cases. We will, therefore, not focus on this approach. However, the utility of SWOW for constructing target embeddings will improve with larger SWOW data sets in the future. Large language models solve the problem of limited coverage, as they can generate embeddings for any string of text in multiple languages. A potential advantage of such embeddings may be that they capture the same information about the targets as provided by the distributions of free association responses (Hussain, Mata, & Wulff, 2024; Hussain, Mata, Newell, & Wulff, 2024). A potential disadvantage of large language models is a lack of transparency and interpretability relative to free associations.

A final point of consideration concerns projections of the embedding space to reduce dimensionality. To visualize the relationships between targets, it is necessary to reduce the embedding to two dimensions. This can be achieved via standard linear methods, such as SVD or principle component analysis. Alternatively, nonlinear approaches can be used, such as multi-dimensional scaling or manifold approximation algorithms like UMAP (McInnes et al., 2018) and PaCMAP (Wang et al., 2021). The latter can sometimes produce more useful projections with greater separation between target locations, but care must be taken not to over-interpret the target placements in such projections (Chari & Pachter, 2023). It should also be noted that approaches like UMAP or PaCMAP have stochastic components that will prevent exact reproducibility if no random seeds are set, and that these algorithms depend on hyperparameters that can greatly influence the outcome (McInnes et al., 2018; Wang et al., 2021).

### Embedding targets with *associatoR*

The ar_embed_targets() function implements different approaches to generating target embeddings for an associatoR object provided as input. To generate response-based embeddings using PPMI and SVD, the function’s method argument must be set to “ppmi-svd”. The optional n_dim argument specifies the number of dimensions of the resulting target embedding. The embedding will be added as an additional element in the associatoR object returned by the function.

**Figure:**
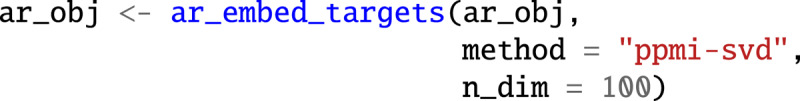

To generate embeddings using large language models from Hugging Face, the method argument must be set to “huggingface”. Additionally, the function requires specification of an access token using the token argument; this can be obtained by creating a free account with the platform. Per default, the sentence-transformer MPNet model^1^ will be used; this model achieves a good compromise between computing needs and performance. Other models can be specified using the model argument. See https://huggingface.co/models for an overview of models.

**Figure:**
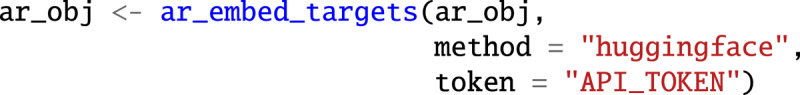

The ar_project_embedding() function projects the target embeddings into a two- or more dimensional space. Three algorithms can be selected via the method argument: principal component analysis (“pca”), multi-dimensional scaling (“mds”), or UMAP (“umap”). The ar_project_embedding() function takes the associatoR object as input and replaces the target embedding with the projection.

**Figure:**
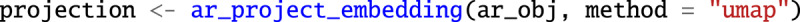

## 5. Characterizing targets

As a final preparatory step before the main free association analysis, targets can be characterized along several dimensions. These include their frequency among the responses, their psycholinguistic properties such as valence or concreteness based on databases (e.g., SCOPE; Gao et al., 2023), and their average response position in studies that collect multiple responses per cue. These characteristics can be useful in subsequent analysis assessing, for instance, which group of individuals retrieves certain targets more often or produces more positive associations.

Another important target characteristic is their cluster grouping, which will require more in-depth discussion. Sorting targets into a small number of clusters can help communicate the complex response patterns inherent to free associations and simplify downstream analyses. Clustering identifies groups of targets with similar embeddings and, by extension, similar meanings. Producing clusters involves transforming the target embedding into a matrix of target similarity and distances and supplying this matrix to an off-the-shelf clustering algorithm. There are many algorithm options, varying in the criteria they seek to optimize and thus in the definition of good clustering (Ezugwu et al., 2022; Haslbeck & Wulff, 2020; Hennig, 2015). Algorithms such as k-means, Gaussian mixture clustering, or the network-based Louvain algorithm focus, in different ways, on minimizing the average distance or maximizing the average similarity within clusters. Other algorithms, such as hierarchical clustering with complete linkage or DBSCAN, focus on minimizing the maximum distance within clusters. There is no reason to generally prefer one algorithm over others; researchers should evaluate the options based on their goals.

A related question is how to select an appropriate number of clusters. Some algorithms (e.g., Louvain and DBSCAN) automatically return a certain number of clusters by optimizing an internal clustering criterion. Alternatively, estimates of the number of clusters can be obtained using established metrics such as the gap statistic or clustering instability (Haslbeck & Wulff, 2020; Tibshirani et al., 2001). While these metrics suggest an ‘optimal’ number of clusters, we recommend against using this approach for the analysis of free associations. Elements in semantic embedding spaces are typically distributed somewhat evenly without obvious thematic borders. They are further organized hierarchically, implying equally justifiable clustering solutions at different levels of granularity. Instead, we advocate viewing target clusters based on cluster analysis as a useful data summary that assists communication and analysis. Exploring different algorithms and numbers of clusters will help to identify an appropriate clustering. However, this process should be communicated transparently. We recommend evaluating the similarity between clusters and the stability of cluster assignments; see Wulff and Mata (2022) for an example.

We discuss the results of producing target characteristics for our working example in the next section on mapping targets, which brings together target embedding and target characteristics to produce a visual overview of responses.

### Characterizing targets with *associatoR*

The ar_count_targets() function evaluates the overall frequency of targets among the responses. It takes an associatoR object as input and adds the frequencies to the targets table.

**Figure:**
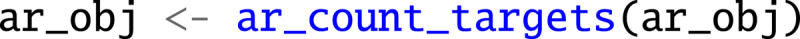

The ar_characterize_targets() function labels targets based on databases of common psycholinguistic word features. It takes the associatoR object as input and adds characteristics specified using the characteristics argument to the targets table. Currently, up to five different word features can be selected, namely valence, arousal, dominance, concreteness, and word frequency, which are obtained from the databases of Warriner et al. (2013) and Brysbaert et al. (2014). Instead of a character vector of implemented features, the function can also take a lookup table of user-specified characteristics as input. Note that if features are unavailable for some targets in preset or user-specified lookup tables, the function returns ‘NA’.

**Figure:**
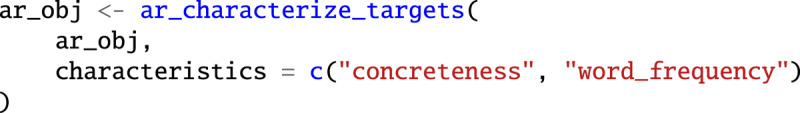

The ar_summarize_targets() function summarizes response characteristics for each target, such as the average response position of each target. It takes the associatoR object, a response_var (here, response_position), and a function (fun; here, mean) as input and adds the response_var summary to the targets table, in the case below, the mean response position for each target.

**Figure:**
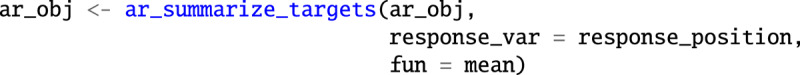

The ar_correlate_targets() function evaluates response frequencies of targets with respect to participant characteristics, such as gender or age. It takes the associatoR object as input, as well as one or more participant variables from the object’s participants table. Further, the function takes a value for the metric argument, which controls how to evaluate the relation between target counts and participant variables. The value “auto” triggers the selection of an appropriate metric based on the scale levels of the participant variables. The resulting correlations are added to the targets table.

**Figure:**
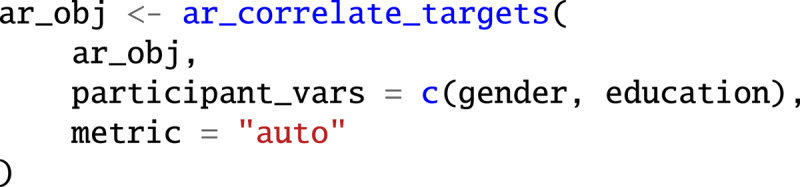

The ar_cluster_targets() function uses clustering algorithms to produce cluster groupings based on the target_embeddings table. The argument method specifies the clustering algorithm to use. The code below implements target grouping using Louvain clustering, which optimizes the modularity of a network based on the cosine similarities between target embeddings (see Blondel et al., 2008; Wulff & Mata, 2022). Additional optional arguments can be used to specify method-specific parameters, such as the number of clusters or resolution.

**Figure:**
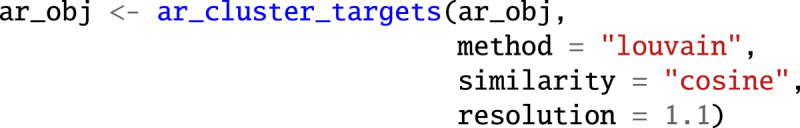

## 6. Mapping targets

One core goal of using free association responses is to understand the breadth of associations that spontaneously come to participants’ minds when they think of a topic or concept. Mapping targets combines the analytic results of previous steps to produce an overview of these associations using visualizations.

**Figure 3:**
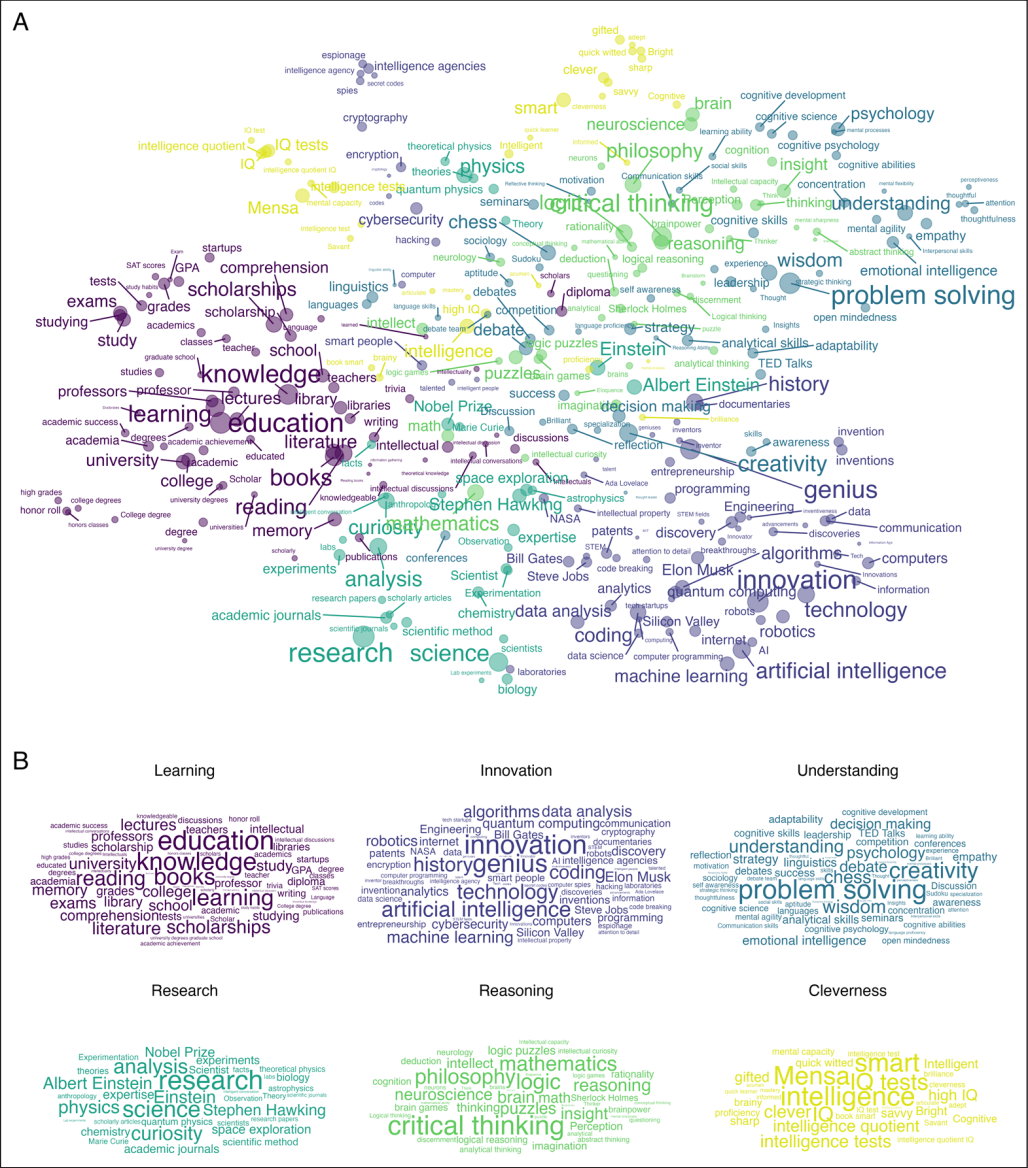
GPT-4-Turbo-generated free associations to the cue word ‘intelligence’. **(A)** A UMAP projection of the response-based embedding determined word positions. Word and point size show response frequency; colors show membership to clusters. **(B)** Word clouds for each cluster, with font size indicating word frequency among the responses.

Figure 3A depicts a two-dimensional UMAP projection of the response-based target embedding. The figure shows points and text labels for the 313 targets with a frequency of at least 5, with points and text scaled on the basis of the target’s response frequencies. The placement of targets reflects the similarity based on the embedding, with similar points generally placed closer in the projection space. Additionally, the targets are colored according to six target clusters identified using Louvain clustering of the target embedding (Blondel et al., 2008). As Figure 3A shows, targets belonging to the same cluster are not always grouped closely together. This results from the nonlinear projection technique, where no strong meaning should be attached to distances between specific target pairs.

Figure 3B illustrates the target clusters as word clouds, with frequent targets displayed in larger font and more centrally. The first cluster features associations surrounding the topic of *Learning*. It contains 22.4% of targets and 24.6% of responses, with the most frequent responses being *education* (2.06%), *knowledge* (1.79%), and *learning* (1.52%). The second cluster features associations surrounding the topic of *Innovation*. It contains 22.7% of targets and 22.4% of responses, including *innovation* (1.99%), *genius* (1.77%), and *artificial intelligence* (1.31%). The third cluster features associations surrounding the topic of *Understanding*. It contains 19.8% of targets and 17.3% of responses, including *problem solving* (1.98%), *creativity* (1.39%), and *wisdom* (0.96%). The fourth cluster features associations surrounding the topic of *Research*. It contains 10.9% of targets and 15.4% of responses, including *research* (2.08%), *science* (1.52%), and *analysis* (1.23%). The fifth cluster features associations surrounding the topic of *Reasoning*. It contains 13.7% of targets and 13.7% of responses *critical thinking* (1.75%), *logic* (1.14%), and *philosophy* (1.07%). The sixth and final cluster features associations surrounding the topic of *Cleverness*. It contains 10.5% of targets and 6.5% of responses, including—alongside the cue ‘intelligence’—*smart* (0.73%), *Mensa* (0.71%), and *IQ tests* (0.57%).

The six clusters displayed in Figure 3B help to understand what GPT-4 (or a comparable sample of humans) sees as relevant components of the concept of intelligence. Whereas past work using manual ratings of open-ended questions identified *knowledge*, which is similar to our cluster *Learning*, and *understanding* as key aspects of people’s mental representation of intelligence (Coane et al., 2023), the clusters highlight four further topics: *innovation, research, reasoning*, and *cleverness*. This demonstrates the power of free associations to reveal the breadth of mental representations.

It is important to remember, however, that the six clusters represent just one way of summarizing the data; others may be equally justifiable. Moreover, not all clusters may represent clearly separable components. One way to document this is by using cluster stability analyses (see Haslbeck & Wulff, 2020; Wulff & Mata, 2022). Such analyses perturb the data and evaluate whether targets are reliably assigned to the same clusters. Based on the reliability of these assignments, it is possible to assess how stable each cluster is and to which other clusters individual targets could also have been assigned. In our working example, the cluster *Innovation* was most stable, with an average proportion of same-cluster assignments of .65, followed by *Learning* (.54), *Reasoning* (.53), *Cleverness* (.48), *Understanding* (.43), and *Research* (.41). These numbers suggest that, across the perturbed datasets, targets were grouped with other targets from the same clusters in 65% to 41% of cases. Considering the chance level of 1/6 ≈ 16.7%, these numbers indicate substantial stability. Nevertheless, there were some notable proportions of different-cluster assignments. These were highest for *Understanding* and *Reasoning* (.29), *Reasoning* and *Cleverness* (.17), and *Innovation* and *Research* (.16), suggesting that these clusters share similarities and could potentially form joint clusters.

Target clusters can further be analyzed in several ways. First, to understand which clusters are at the top of respondents’ minds, the clusters can be characterized with respect to retrieval position. In our working example, the targets in the *Cleverness* cluster were retrieved earliest (average position 2.71), followed by *Reasoning* (2.93), *Innovation* (3.04), *Research* (3.06), *Learning* (3.10), and *Understanding* (3.14). Second, to evaluate the contents of clusters, the clusters can be characterized on psycholinguistic dimensions, such as valence. In our working example, the targets in the *Cleverness* cluster were most positive (average valence 6.72), followed by *Learning* (6.51), *Understanding* (6.25), *Innovation* (6.15), *Reasoning* (5.93), and *Research* (5.90) (rated on a scale from 1 to 9; Warriner et al., 2013). Finally, the clusters can also be analyzed with respect to individual difference factors. This will be the focus of the next section.

### Mapping targets with *associatoR*

The *associatoR* package provides two functions to create the above visualizations. The ar_plot_embedding() function creates a plot as shown in Figure 3A. The function takes the associatoR object as input, positions points and labels based on the first two dimensions of the target_embeddings, ideally generated using ar_project_embedding(). Points and labels are sized according to the target frequencies among the responses. Using the argument color_by, the points and labels can further be assigned colors according to another variable; here we use the cluster variable. The ar_plot_embedding() function returns a ggplot2 object that can be extended using other functions of the *ggplot2* ecosystem.

**Figure:**
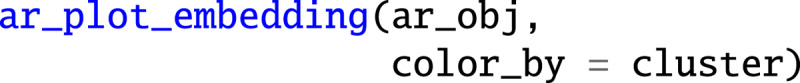

The ar_plot_wordcloud() function creates wordclouds as shown in Figure 3B. It permits specification of colors analog to ar_plot_embedding() and additionally facet_col and facet_row arguments to optionally separate word clouds into rows and columns based on variables in the data; in this case, columns by target clusters. The ar_plot_wordcloud() function also returns a ggplot2 object, permitting further modification and extension.

**Figure:**
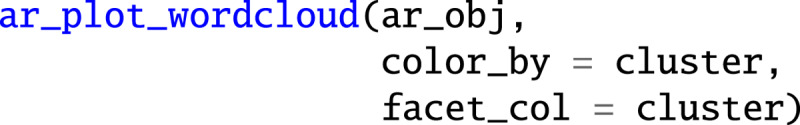

The ar_cluster_stability() function evaluates the stability of a given clustering solution with respect to random perturbations in the embedding. It draws repeated bootstrap samples from the set of targets, reruns the clustering, and evaluates for every pair of targets the average proportion of being placed in the same cluster. Based on these proportions, the function returns a list with two tabular outputs. The first element shows the probability of targets being grouped together in the cluster for all pairs of targets. The second element aggregates this information and shows the probability of being grouped together at the cluster level. This second element allows for an assessment of clustering stability. The diagonal of this element indicates the stability of a cluster, which ideally is 1, indicating that all targets of a cluster are grouped strictly together across all bootstrap samples. The off-diagonal entries, by contrast, show how often targets of one cluster are grouped with targets of other clusters, which can be used to assess cluster similarity or independence. See Haslbeck and Wulff (2020) for more theoretical background on clustering stability.

**Figure:**
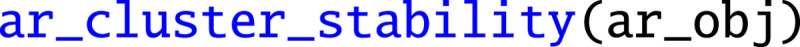

## 7. Comparing targets

A powerful feature of free associations is that they not only provide a rich aggregate overview of topic-related mental representations but also offer insights into how these vary between groups of individuals. Group comparisons can be carried out at three levels: the representation, cluster, and target levels.

At the representation level, embeddings can be constructed separately as a function of individual difference factors, such as gender or education, and then compared in terms of their similarity. This approach, called representational similarity analysis, has previously been employed to study differences in semantic networks across age groups (Wulff, Hills, & Mata, 2022) and is common in neuroscience and research on deep neural networks (Sucholutsky et al., 2023). Representational similarity analysis involves computing similarity matrices, typically using cosine similarity, based on group-specific target_embeddings and comparing the similarity matrices using Spearman correlation. In the context of free association analysis, evaluating representational similarity can reveal which individual difference factor has the greater influence on the composition of mental representations.

When comparing the representational similarity of the four groups in our working example, we observed the highest correlation between the male high school-educated and female high school-educated groups (*r* = .45), followed by the male high school-educated and male university-educated groups (*r* = .43). The lowest correlation was between the male high school-educated and female university-educated groups (*r* = .36). Both individual difference factors seem to have similar effects on mental representations individually (gender-specific groups *r* = .34, education-specific groups *r* = .32).

**Figure 4:**
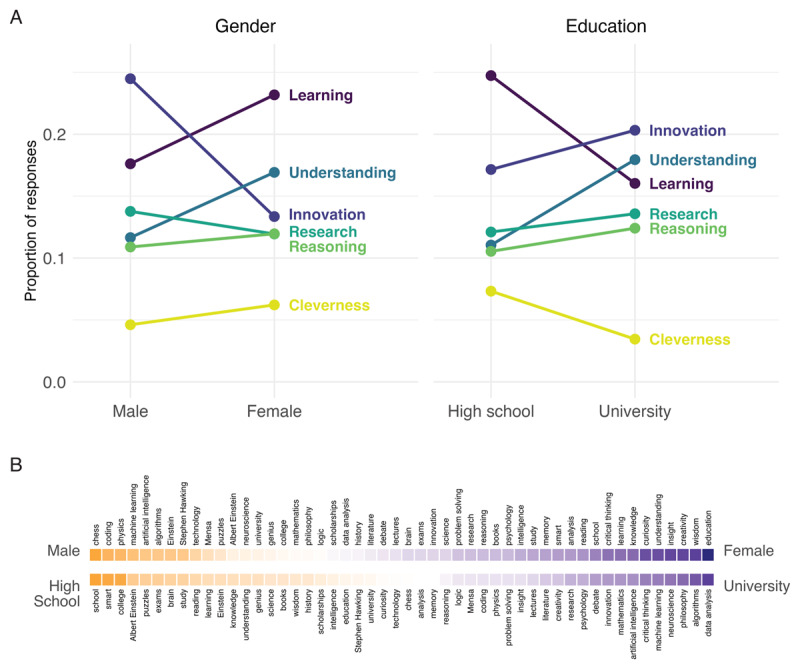
Comparisons between demographic groups: **(A)** Comparison of cluster proportions between male and female (high school- and university-educated) simulated subjects. **(B)** Top 50 high-frequency words among the responses ordered by phi coefficient between male vs. female (high school-vs. university-educated) simulated subjects from more frequent among males (high school-educated) on the left to more frequent among females (university-educated) on the right.

At the cluster level, the response frequencies for each target cluster can be counted as a function of the participant group. This can reveal how the relevance of target clusters differs between groups. In our working example, we compared the proportions of responses falling into each of the six target clusters as a function of gender and education. The results, shown in Figure 4A, reveal that synthetic male participants retrieved targets belonging to the clusters *Innovation* and *Reasoning* more frequently than synthetic female participants did, whereas the opposite was true for the other four clusters. Furthermore, synthetic high school-educated participants retrieved targets belonging to the clusters *Learning* and *Cleverness* more frequently than synthetic university-educated participants, whereas the opposite was true for the other four clusters. Generally, however, the proportions of responses from the different clusters were, with some exceptions, relatively stable across group levels, consistent with past work by Wulff and Mata (2022).

Finally, at the target level, individual difference factors can be directly related to the frequency of retrieving individual targets. This analysis must be constrained to high-frequency responses for reliability reasons but can nevertheless be useful to accentuate the patterns of results observed at the cluster level. Figure 4B shows the phi coefficient, a common correlation coefficient for binary data, for the relation between retrieving or not retrieving each of the 50 most frequent responses and the two individual difference factors. This shows that synthetic male participants were considerably more likely than synthetic female participants to retrieve *chess, coding*, and *physics*. The opposite was true for *education, wisdom*, and *creativity*. Furthermore, synthetic high school-educated participants were considerably more likely than university-educated participants to retrieve *school, smart*, and *college*. The opposite was true for *data analysis, algorithms*, and *philosophy*. These results generally align with the aggregate results shown in Figure 4B but present a finer picture. For instance, *chess* was more frequently retrieved by synthetic male than female participants, although it belongs to the cluster *Understanding*, which is more prominent among female than male participants. Similarly, the response *understanding* is more often retrieved by high school-educated participants, although it belongs to the cluster *Understanding*, which is more prominent among university than high-school participants. Past work has shown how similar target-level analyses can reveal important social, biological, and cultural circumstances of participants. For instance, in Wulff and Mata (2022), men were considerably more likely than women to name *war* as an associate of risk, whereas the opposite was true for *pregnancy*.

These differences and correlations in Figures 4A and 4B could further be evaluated using the tools of statistical inference, for instance, chi-square tests of statistical independence. We refrain from reporting such results, as the goals of our analysis are primarily nonconfirmatory (Lakens, 2024).

### Comparing targets with *associatoR*

The *associatoR* package provides functions to conduct analyses at all three levels (target, cluster, and representational). At the target level, we have previously presented the ar_correlate_targets() function, which produces the correlations displayed in Figure 4B.

The ar_cross_targets() function generates cross-tables of target response frequencies between discrete variables in targets and participants. This function can be used, for instance, to evaluate the proportion of responses from each target cluster for different individual difference factors. It takes the associatoR object as input and returns a table of grouped statistics. Per default, the function produces frequencies, but the user can request group-normalized counts using the normalize argument.

**Figure:**
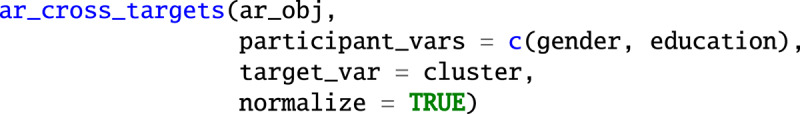

The ar_compare_targets function is analogous to ar_cross_targets(), but for continuous target variables. It summarizes continuous variables, such as psycholinguistic properties of the targets, taking into account the target response frequencies. As inputs, the function takes the associatoR object, participant and target variables of interest, and a summarizing function. It returns a table containing grouped statistics.

**Figure:**
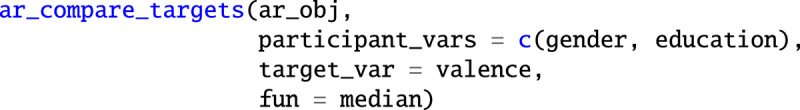

The ar_compare_embeddings function evaluates the representational similarity of target_embeddings for each of one or more variables in the participants table. The group-specific target_embeddings are generated in the background. It calculates the representational similarity based on either the correlation of the lower-triangle similarity matrices or the average correlation of row similarities. This can be specified via the type argument. The function permits passing arguments to ar_embed_targets() in the background via the ellipsis argument.

**Figure:**
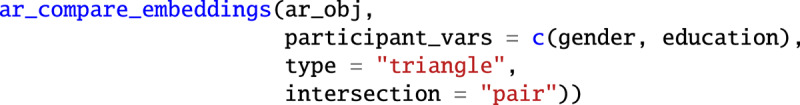

## Discussion

We presented a tutorial demonstrating how free association responses can be collected, processed, mapped, and compared across groups using the R package *associatoR*. Despite a long history in psychology, free association remains an underutilized technique for mapping mental representations of concepts and topics relevant to many research questions and domains. Our tutorial covered the whole analysis workflow, from acquiring and preprocessing data to producing a visual overview of responses and comparing associations between groups of individuals. We showcased these steps using a synthetic data set and the *associatoR* package, which was specifically built for free association analysis.

As an assessment tool, free association offers a unique combination of strengths. It is open-ended, directly taps into what is at the top of people’s minds, affords various analytical approaches, can uncover topics unanticipated by researchers and be applied at scale. It can thus provide novel perspectives on how people think about various topics, enriching the academic discourse in the cognitive and social sciences. Currently, self-report surveys and controlled experimental tasks dominate the empirical methodological landscape in these fields. However, these methods may fail to capture important aspects of human psychology. Surveys may omit relevant items. Behavioral tasks may lack ecological validity, leading to poor generalizability. Both can be misconstrued by participants in various ways (Avis et al., 2014; Szollosi & Newell, 2020). An illustrative example of the limitations of these approaches is research on risk, where abstract behavioral tasks hardly correlate with related behavioral tasks or real-world observations, and self-report items (and behavioral tasks) may fail to consider how people interpret risk and risk-taking differently (Hertwig et al., 2019; Mata et al., 2018; Wulff & Mata, 2022). These limitations could be partially addressed by using qualitative research methods, such as interviews, to help uncover how people interpret and process both psychological measures and real-life situations differently depending on their accumulated experience and sociocultural circumstances. However, qualitative approaches can be laborious and prone to subjective biases. Free association can fill a gap in the empirical toolbox of cognitive and social scientists by revealing, at scale, how people think about concepts, situations, and other aspects of the world.

We believe that our example illustrates several further strengths of the free association approach. First, free associations can help reveal laypeople’s understanding of central psychological constructs, such as intelligence (Sternberg et al., 1981). Second, free association can be effective as an opinion-gauging tool, helping to understand the diversity of opinion on many real-world issues and across various demographic groups. In our synthetic example, we created groups based on gender and education. The analysis pipeline can be extended to examine the role of political affiliation or other demographics, thus helping to document polarization and inform strategies that combat it (File et al., 2019). Similarly, free association can provide insights into different groups’ thinking on companies or products, thus helping to document consumer preferences and inform marketing efforts (Koll et al., 2010), or reveal differences in representations between experts and students in learning contexts that may help to design more effective educational programs (Stella et al., 2019). Third, as shown in our example, free associations can be employed to study representations of machine systems with human-like language interfaces, such as large language models, ultimately contributing to the advancement of research on human–machine representational and behavioral alignment (Sucholutsky et al., 2023).

Several limitations of the free association approach also warrant discussion. First, insights obtained from free associations remain abstract, and it is difficult to connect them to human attitudes or behavior in concrete situations. Empirical evidence suggests weak to moderate links between free associations and other forms of assessment, including self-reports and behavioral tasks (Schnabel & Asendorpf, 2013; Wulff & Mata, 2022). Second, being open-ended, free associations can present challenges to standardization across individuals, with implications for interindividual comparison. As a result, work using free association is likely best geared to group comparisons rather than to assessment of individual differences. Third, there is currently a lack of empirical evidence to inform the choice of several design features, including number of participants, number of responses per cue, and number of response levels. In past work, choices for these parameters were based on research that focused on measuring the structure of semantic networks (e.g., the decision to use three responses per cue was based on the approach taken in the citizen science SWOW study). Further empirical and simulation work should be conducted to help inform design choices that make free associations even more effective in mapping the mental representations. Fourth, with respect to our analysis of GPT-4’s understanding of intelligence, it is important to highlight that results can drastically depend on the prompt used to generate the responses (see Appendix). This particularly concerns the demographic steering used to generate responses for different levels of gender and education. Future work should clarify how to best generate free associations from large language models to investigate the representations of topics, including demographic biases (Atari et al., 2023).

## Conclusion

Researchers and policymakers alike can benefit from a clearer picture of how people think about key topics such as risk, artificial intelligence, and politics. Here, we have demonstrated how free associations can be used to map out the complexity and variability of people’s thinking, showcasing the workflow with a synthetic data set generated by GPT-4. We envision that such analyses will enrich the methodological repertoire of the cognitive and social sciences and hope that this tutorial and the *associatoR* R package will make free association analyses more accessible, reproducible, and effective.

## Data Accessibility Statement

The data used in this analysis is available at https://github.com/samuelae/GPT4-intelligence/blob/48198d09ef2c60ce0b03bd59afe4a79ad293e8a6/01_Data/intelligence_associations.csv. Additional information on data generation can be found in the Appendix.

## Footnotes

see https://huggingface.co/sentence-transformers/all-mpnet-base-v2.

## Appendix

### GPT-4 Intelligence Associations

We used the OpenAI API to generate our *intelligence* data set with the model “GPT-4-0125-Preview”. This data set simulates 1,000 subjects providing five free associations to the cue word ‘intelligence’ (first level responses), and an additional five free associations to each of their prior responses (second level responses). In this Appendix, we report the prompts used and describe the post-processing steps.

### Prompt

We used the following prompt to generate the *intelligence* free association data ({gender}, {education}, and {cue} replaced by the respective gender, education, and cue words): “What are the first five associations that come to a {gender} {education}-educated adult’s mind when thinking of the cue ‘{cue}’? Produce responses highly typical for a {gender} {education}-educated adult. Separate the responses by comma. Produce ten separate sets of five responses separated by semicolon.”

### Processing

We generated our data in multiple steps. First, we generated all first-level responses, followed by data cleaning and normalization to ensure high-quality labels for subsequent simulation. Second, we generated second-level responses to each of the first-level responses, making sure to produce them within the same system prompt runs as for the first-level responses, followed by the same cleaning and normalization process as for the first-level responses. Third, we combined the first-level and second-level responses to simulate 250 subjects from each combination of demographic levels.

### Code and data availability

The code to produce this data set as well as the data can be accessed on GitHub (https://github.com/samuelae/GPT4-intelligence). The resulting *intelligence* data set is also available in the *associatoR* R package. Please note that the generation of free associations using OpenAI’s API will be charged based on the number of tokens processed. Further, due to the nondeterministic nature of this data set generation, reproducing the data will not result in the exact same data set.
